# Efficient airborne transmission of influenza D virus in ferret models and serological evidence of human exposure in Northeast China

**DOI:** 10.1080/22221751.2025.2564308

**Published:** 2025-10-10

**Authors:** Hongbo Gao, Weiyang Sun, Pengyang Lu, Zhipeng Dong, Jiajing Wu, Yuanguo Li, Wentao Wan, Yue Feng, Bingshuo Qian, Mingzhu Zhang, Yafei Wu, Chunling Dong, Beilei Shen, Tiecheng Wang, Xianzhu Xia, Jie Zhang, Wuchun Cao, Yuwei Gao

**Affiliations:** a Changchun Veterinary Research Institute, Chinese Academy of Agricultural Sciences, State Key Laboratory of Pathogen and Biosecurity, Key Laboratory of Jilin Province for Zoonosis Prevention and Control, Changchun, People’s Republic of China; b Henan Provincial Engineering Center for Tumor Molecular Medicine, School of Basic Medical Sciences, Henan University, Kaifeng, People’s Republic of China; c College of Veterinary Medicine, Shanxi Agricultural University, Jinzhong, People’s Republic of China; d School of Pharmacy, Henan University, Kaifeng, People’s Republic of China; e State Key Laboratory of Pathogen and Biosecurity, Beijing, People’s Republic of China; f Department of Respiratory and Critical Care Medicine, The Second Hospital of Jilin University, Changchun, People’s Republic of China

**Keywords:** Influenza D virus, airborne transmission, seropostivity, silent spread, drug susceptibility

## Abstract

Newly emerging influenza D virus (IDV), first identified in swine in 2011, has demonstrated broad mammalian tropism with notable prevalence in bovine populations and occupational exposure-associated seroprevalence among cattle workers. This zoonotic expansion raises concerns that IDV could acquire capability for human-to-human transmission via sustained evolving in mammal hosts. Here, we evaluated the infectivity and transmissibility of a currently circulating IDV strain, D/bovine/Jilin/HY11/2023 (abbreviated as D/HY11), isolated from cattle in Northeast China in 2023. D/HY11 was able to replicate efficiently in human primary respiratory epithelial cells and exhibits respiratory tract tropism in mammals. More importantly, we found that D/HY11 could efficiently transmit through the air between ferret models (5/6). Serological surveillance (2020–2024) revealed alarming exposure rates, with no significant difference in positivity between rural and urban populations: 73.37% (449/612) in the general population and an even higher rate of 96.67% (58/60) among individuals with respiratory symptoms. The extraordinary high IDV seropositivity among people in Northeast China highlights the possibility of silent spread in mammals with mild symptoms. Among generic anti-influenza drugs tested in vitro, only polymerase inhibitors demonstrated effective suppression of IDV replication. And the D/HY11 strain exhibited enhanced polymerase activity compared to the classical IDV strain, with preliminary evidence implicating the P3 gene as a potential contributing factor to this functional enhancement. Our pathogenetic and serological findings indicate that IDV may have acquired the capacity for human-to-human transmission during its ongoing evolution, and currently circulating IDV strains already pose a potential panzootic threat.

## GRAPHICAL ABSTRACT

**Figure:**
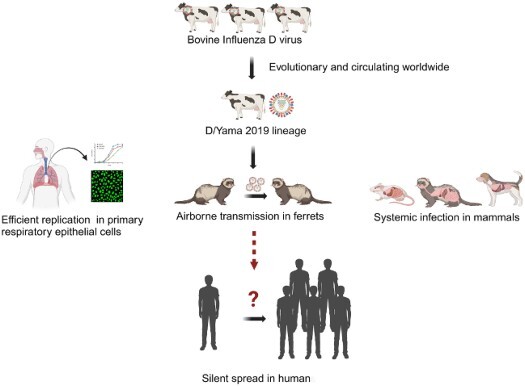

## Introduction

Influenza D virus (IDV) is a novel RNA pathogen belonging to the family Orthomyxoviridae, first identified in 2011 in a pig exhibiting influenza-like symptoms in Oklahoma [1]. Cattle were subsequently recognized as the primary host for IDV [2]. A seroprevalence study among cattle workers in Florida found that up to 95% of individuals with occupational exposure to cattle tested positive for anti-IDV antibodies [3], suggesting potential zoonotic transmission through occupational contact. Most experimental studies on mammalian infections have demonstrated direct contact transmission, with no evidence of efficient airborne transmission to other hosts except calves [1,4–6]. However, in recent years, IDV often arrives silently in a new country or continent and does not display symptoms, with both virological and serological evidence of various countries including Americas, Europe, Asia and Africa [7–11], and multiple species such as small ruminants (goat, sheep), horses, camels, and dogs [12–15]. This raises concerns about whether IDV has acquired enhanced infectivity and transmissibility.

The ability of a novel influenza virus to cause disease and transmit through the air is a critical characteristic of pandemic influenza strains [16]. Consequently, understanding the inherent virulence and transmissibility of emering IDV strains is crucial for pandemic preparedness. Ferrets can transmit human influenza viruses to other ferrets with or without direct contact, making them a valuable model for assessing the zoonotic risk of animal-derived influenza strains [17]. While limited airborne transmission of H5N6, H7N9, H10N3, and bovine H5N1 viruses has been observed in mammalian models [18–21], the H1N1 pdm09 virus demonstrated efficient transmission in these models [22]. Efficient spread in ferret model suggests a potential for human transmission.

Here, we tested the replicative capacity and pathogenicity of a bovine IDV strain isolated in Northeast China in October 2023 [23], using primary respiratory epithelial cells and mammalian animal models, respectively. Furthermore, its transmissibility in ferret models was also investigated. Our results demonstrated that this novel IDV strain exhibited efficient transmission between ferrets not only by direct contact but also through the air. To assess potential human exposure, we conducted serological surveillance among people in Northeast China, measuring serum antibody levels against IDV. Furthermore, the susceptibility of IDV to common anti-influenza drugs and its polymerase activity were evaluated.

## Materials and methods

### Cells, viruses, and samples

Madin-Darby Canine Kidney (MDCK) cells and Human embryonic kidney HEK-293 T cells were obtained from the American Type Culture Collection and cultured in Dulbecco’s modified Eagle medium (DMEM; Gibco, USA) supplemented with 10% fetal bovine serum (FBS; Gibco, Australia) and 1% antibiotic-antimycotic (Gibco, USA). Well-differentiated primary airway epithelial cell (AEC) cultures, including human nasal epithelial cells (HNECs), human tracheal epithelial cells (HTEpic), human alveolar epithelial cells (HAECs), bovine nasal epithelial cells (BNECs), bovine tracheal epithelial cells (BTEpics), swine nasal epithelial cells (SNECs), swine tracheal epithelial cells (STEpics), canine nasal epithelial cells (CNECs), and canine tracheal epithelial cells (CTEpics) were purchased from Cellverse Bioscience Technology Co., Ltd., (Shanghai, China) and maintained in specific growth media containing 2% FBS and 1% antibiotic-antimycotic. All cells were cultured at 37°C in a 5% CO₂ humidified atmosphere.

The bovine IDV strain D/bovine/Jilin/HY11/2023 (abbreviated as D/HY11) was isolated from cattle in our previous study [23]. The human seasonal H1N1 influenza A virus strain A/Hebei/F076/2018 (abbreviated as A/F076) was used as a control [24]. All viruses were propagated and titrated in MDCK cells in DMEM containing 2 µg/mL L-1tosylamide-2-phenylethyl chloromethyl ketone (TPCK)-treated trypsin (Biochemical, USA) at 37°C for 72 h.

Archived human serum samples were anonymously obtained from hospital serum banks in Northeast China, along with information on the presence of respiratory symptoms and the year of sampling.

### Tissue Cultured Infectious Dose 50 (TCID_50_) determination

Virus titres were determined by TCID_50_ assay in MDCK cells using 96-well plates. To quantify peak viral titres, tenfold serial dilutions of virus were prepared in virus culture medium and inoculated onto confluent MDCK cell monolayers in 96-well plates (three replicates per dilution) for 1.5 h. After adsorption, the inoculum was removed and replaced with maintenance medium consisting of Opti-MEM (Gibco, USA) supplemented with 2 µg/mL TPCK-treated trypsin and 1% antibiotic-antimycotic. The plates were incubated at 37°C with 5% CO_2_ in a humidified atmosphere for 72 h, followed by hemagglutination (HA) assay to assess viral growth. Viral titres (TCID_50_/mL) were calculated using the Reed and Muench method [25].

### Growth kinetics of IDV in primary respiratory epithelial cells

We characterized the growth kinetics of D/HY11 in various well-differentiated primary AEC cultures, including human (HNECs, HTEpics, HAECs), bovine (BNECs, BTEpics), swine (SNECs, STEpics), and canine (CNECs, CTEpics) nasal and tracheal epithelial cells. Cells were infected at a multiplicity of infection (MOI) of 0.1. After 1.5 h of incubation at 37℃, the virus inoculum was aspirated, and the cell surface was washed three times with PBS. The inoculum was then replaced with maintenance medium (Opti-MEM, Gibco, USA) supplemented with 0.5 µg/mL TPCK-treated trypsin and 1% antibiotic-antimycotic. The plates were incubated at 37℃ in a humidified 5% CO_2_ atmosphere. Culture supernatants were collected at 12, 24, 48, 72, and 96 h post-infection (hpi) and stored at −80℃ for further analysis. Viral titres were determined by TCID_50_ assay in MDCK cells. All experiments were performed in triplicate.

### Immunofluorescence microscopy

Prior to infection, well-differentiated monolayer primary AEC cultures were gently washed three times with warm PBS to remove mucus deposits. Cells were infected with specified viruses at an MOI of 0.1 and incubated at 37°C for 48 h. Cultures were fixed in 4% formaldehyde (Beyotime, Beijing, China) at 48 hpi and stained for immunofluorescence as previously described [26]. After fixation for 2 h, the cultures were permeabilized with 0.2% Triton X-100 (Beyotime, Beijing, China) for 30 min. After blocking with 5% bovine serum albumin (Beyotime, Beijing, China) for 2 h at 37°C, cells were incubated overnight at 4°C with rabbit abbit anti-influenza nucleoprotein (NP) antibody (1:1000; Sino Biological, Beijing, China), which targets the prototypic D/bovine/Oklahoma/660/2013 strain. This custom antibody has been previously validated for immunofluorescence assays, as commercial IDV-specific antibodies are currently unavailable [26]. Subsequently, cells were incubated with fluorescein-isothiocyanate (FITC)-labeled goat anti-rabbit IgG secondary antibody (1:1000; ab6717, Abcam, Beijing, China) for 1 h at room temperature. For ciliated cell identification, nuclei were counterstained with 4′,6-diamidino-2-phenylindole (DAPI; Beyotime, Beijing, China). Fluorescent images were acquired using a APEXVIEW APX100 fluorescence microscope (Olympus, Japan).

### Mouse infection study

Female BALB/c mice (three- and six-week-old) were obtained from Vital River Laboratory Animal Technology (Beijing, China) and housed in individually ventilated cages under specific pathogen-free conditions. Six-week-old mice were randomly divided into two groups: control group (n = 6, PBS-inoculated) and infected group (n = 15, virus-inoculated). Three-week-old mice were similarly allocated into control (n = 6) and infected (n = 30) groups. Following light anesthesia with isoflurane, mice were intranasally inoculated with 10^6^ TCID_50_ of virus in a 50-µL volume. Control animals received an equal volume of PBS alone. Clinical signs were monitored and body weights were recorded daily. Any animal showing a weight loss greater than 30% or signs of suffering was humanely euthanized in accordance with ethical guidelines.

In six-week-old mice, subgroups (n = 3) were humanely euthanized at 3, 5 and 7 days post-infection (dpi) for collection of nasal turbinate and lungs for viral titration. Blood samples were collected at 3, 5, 7, and 14 dpi for serum preparation.

Three-week-old mice (n = 6 per time point) were euthanized at 2, 4, 6, and 8 dpi for comprehensive tissue sampling (nasal turbinate, trachea, lungs, brain, heart, liver, spleen, kidneys, and intestines) for viral load determination. Additionally, lungs and trachea were collected for histopathological analysis. Terminal blood collection was performed at 14 dpi for serological studies.

### Canine infection study

Two-month-old IDV-seronegative beagles were purchased from a certified breeding facility in Shenyang, China. Dogs in the experimental group were anesthetized with Zoletil 50 (Virbac, France) and inoculated intranasally with 10^6^ TCID_50_ of D/HY11 in a volume of 1 mL. Control animals received an equal volume of sterile PBS. Clinical monitoring included daily measurements of body weight, rectal temperature, and observation of clinical signs throughout the 14-day study period. Nasal, throat, and rectal swabs were collected daily from days 1–8 dpi, with blood samples obtained at 14 dpi for serological analysis.

For pathological evaluation, two infected dogs and one control animal were humanely euthanized at 3 dpi. To quantify IDV, tissues were frozen, homogenized and subjected to TCID_50_ assay and RNA extraction. Another set of tissues was fixed in 10% neutral buffered formalin, paraffin-embedded, and sectioned for hematoxylin and eosin (H&E) and immunohistochemistry (IHC) assays. Hemagglutination inhibition (HI) assays were performed to determine serum antibody levels.

### Ferret transmission and infection study

Four-month-old IDV-seronegative female ferrets were purchased from Wuxi Cay Ferret Farm (Jiangsu, China). All animals were housed in wire cages within high-efficiency particulate air filter isolators under biosafety level 2 conditionsAll ferrets were acclimatized for 7 days under controlled conditions prior to experimentation, with ad libitum and access to water and standard diet, and maintained on a 12-h light/dark cycle. The direct-contact and airborne-contact transmission experiment in ferrets was performed as described previously [27]. For each transmission model (n = 3 ferrets/group), animals were anesthetized with a combination of Zoletil 50 (1 mg/kg; Virbac, France) and xylazine (1 mg/kg), then intranasally inoculated with 10⁶ TCID₅₀ of virus in 0.5 mL volume. The following day, each infected ferret was co-housed with a naive contact ferret; another ferret was also housed in a wire-frame adjacent cages (5 cm apart) to the infected ferret, allowing only aerosol transmission (Figure 3A). In further airborne-contact transmission studies, the inoculated ferret was placed in a cage, and one day later, a naive ferret was placed in an adjacent cage (5 cm apart) (Figure 3B). Viral shedding was monitored by collecting nasal washes every other day for 14 days, with titres determined in MDCK cells. All ferrets were monitored daily for body weight, temperature, and clinical signs [28]. Serum samples collected at 14 dpi were analyzed for seroconversion by HI assay using chicken erythrocytes. The transmission study was carried out under controlled conditions of 20–25℃ and relative humidity of 40%-60%. All ferrets were not involved in other experimental procedure.

For viral tissue tropism studies, additional ferrets (n = 3) were inoculated as above and euthanized at 4 dpi. Multiple organs (nasal turbinate, trachea, lungs, spleen, kidneys, liver, heart, intestines, and brain) were collected for viral titration. Lung and trachea samples were collected for histopathological examination.

### H&E and IHC analysis

Tissues were fixed in 10% neutral buffered formalin, embedded in paraffin, and sectioned at 3 µm. Sections were stained with H&E following standard procedures to assess tissue architecture and cell morphology. For viral antigen detection, IHC was performed using an in-house rabbit polyclonal primary antibody directed against influenza NP (1:1000, customized by Sino Biological, Beijing, China). A horse radish peroxidase (HRP) conjugated secondary antibody was applied, and colour development was achieved using an HRP reaction kit (ZSGB-BIO, Beijing, China).

### RNA extraction and one-step quantitative PCR

Viral RNA was extracted with a viral RNA purification kit (Bioflux, Hangzhou, China) according to the manufacturer’s protocols. To quantify viral PB1 gene copies, one-step quantitative PCR (Taraka, Japan) was performed on the RNA samples using established methods [29]. Viral PB1 gene plasmids served as standards for curve generation.

### Sequencing

Inoculums and nasal wash specimens collected from ferrets in transmission studies were subjected to whole-genome sequencing. Viral RNA was extracted using the viral RNA purification kit (Bioflux, Hangzhou, China), followed by reverse transcription PCR (RT–PCR) with the HiScript II One-Step RT–PCR kit (Vazyme, Nanjing, China) and previously published primers [23]. PCR products were purified using the NucleoSpin Gel and PCR Clean-up Kit (Macherey-Nagel, Germany). Sanger sequencing was performed on an Applied BiosystemsTM 3730XL DNA Analyzer (Thermo Fisher, China), and the resulting sequences were aligned with reference D/HY11 strain.

### HA and HI assay

HA and HI assays were performed as previously described [30]. Briefly, sera were treated with receptor-destroying enzyme (Denka Seiken Co., Tokyo, Japan) at a 1:4 ratio and incubated at 37°C for at least 18 h, followed by heat inactivation at 56°C for 30 min. The inactivated serum was then diluted to a final concentration of 1:10 in PBS. HA and HI assays were performed using D/HY11 strain and 0.5% chicken red blood cells at room temperature.

### Microneutralization (MN) assay

The MN assay was performed as previously described [31]. Briefly, MDCK cells were cultured at 37°C in 5% CO_2_ and seeded in 96-well plates. Serum samples, heat-inactivated at 56°C for 30 min, were serially diluted two-fold in PBS and mixed with an equal volume of virus (100 TCID_50_/well). After 1 h of incubation at 37°C in 5% CO_2_, the virus-serum mixture was added to 96-well plates with MDCK cells. Following 72 h of incubation at 37°C in 5% CO_2_, HA activity in the supernatant was assessed.

### Drug susceptibility testing

The susceptibility of viruses to baloxavir acid (the active form of baloxavir), favipiravir, amantadine, oseltamivir acid (the active form of oseltamivir), zanamivir, and peramivir was determined by a virus yield reduction assay in MDCK cells, as previously described [32]. All compounds were purchased from MedChemExpress (USA) and dissolved in 100% dimethyl sulfoxide (DMSO; Sigma, USA) to prepare 100 mM stock solutions for in *vitro* testing. Confluent MDCK monolayers in 6-well plates were infected with viruses at an MOI of 0.1 per well. After 1.5 h of adsorption at 37°C to allow efficient viral binding, the virus inoculum was removed. Subsequently, virus culture media containing serial dilutions of baloxavir acid, favipiravir, amantadine, oseltamivir acid, zanamivir, or peramivir supplemented with 2 µg/mL TPCK-treated trypsin and 1% antibiotic-antimycotic were added to each well in triplicate. Supernatants from IDV and Influenza A virus (IAV) infections were collected at 72 and 48 hpi, respectively, and viral titres were determined in MDCK cells. Virus-free samples served as negative controls, and drug-free samples served as positive controls.

### Polymerase activity assay

Polymerase activity was assayed using a mini-genome reporter system in HEK 293 T cells, as previously described [33,34]. The genome sequences of D/swine/Oklahoma/1334/2011were downloaded from the NCBI Genbank database and aligned using MEGA XI. The PB2, PB1, NP, and P3 genes from D/HY11 and D/OK/1334 strains were individually cloned into the pCAGGS plasmid. HEK-293 T cells were co-transfected with PB2, PB1, NP, and P3 expression plasmids (125 ng each), along with the firefly luciferase reporter plasmid p-NP-Luci (10 ng), and internal control pRL-TK (2.5 ng), at the indicated temperature, using Lipofectamine 3000 (Invitrogen/Thermo Fisher, USA) according to the manufacturer’s protocol. Negative controls consisted of p-NP-Luci, pRL-TK and empty pCAGGS plasmid (lacking influenza virus genes). At 32 h post-transfection, cell lysates were prepared using the Dual-Luciferase Reporter Assay System (Promega, Madison, WI, USA), and luciferase activity was measured using a GloMax 96 Microplate Luminometer. Three independent experiments were performed.

### Data and statistical analyses

All data represent the average of at least three independent experiments and expressed as the mean ± standard deviation (SD), as indicated in the figure legends. Statistical analyses were performed using GraphPad Prism 6 with one-way or two-way ANOVA, as appropriate. A *P*-values of <0.05, < 0.01, < 0.001, and <0.0001 were considered statistically significant.

## Results

### Bovine IDV replicates efficiently in primary respiratory epithelial cells

**Figure 1.**
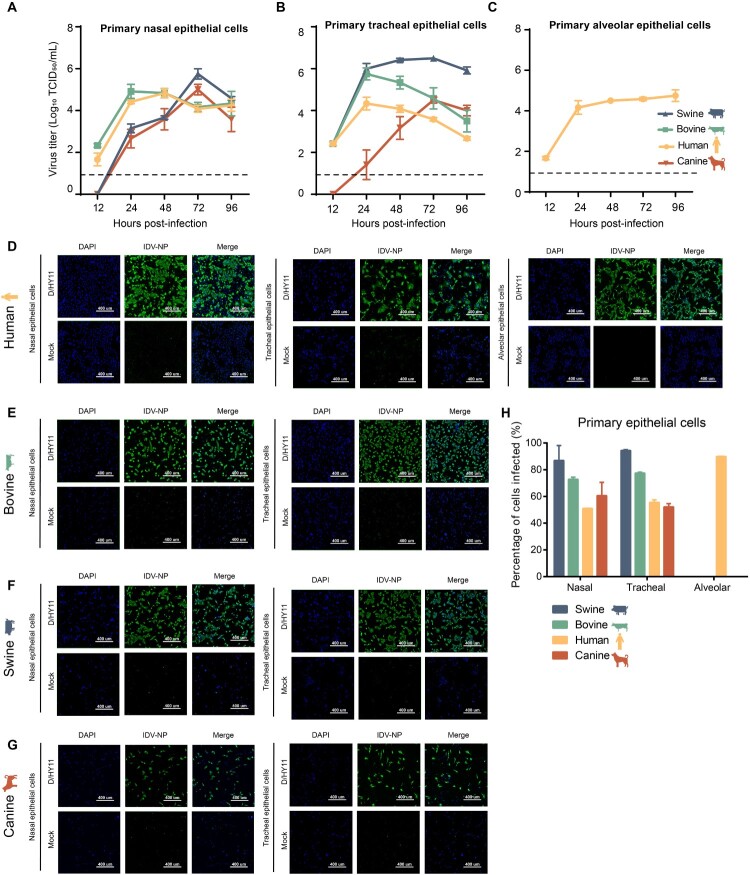
IDV infection of and replication in different primary respiratory cells. Viral growth kinetics of D/HY11 strains in primary respiratory epithelial cells derived from swine, bovine, human, and canine sources following infection at an multiplicity of infection (MOI) of 0.1. Viral titres in nasal (A), tracheal (B), and alveolar (C) epithelial cells were determined by TCID_50_ assays on MDCK cells using cell culture supernatants collected at specified time points. Data are presented as mean log_10_TCID_50_/mL ± SD from duplicates from three independent donors. Dashed lines indicate the lower limit of virus detection. For immunofluorescence analysis, primary respiratory epithelial cells were infected with D/HY11 strains (MOI = 0.1). At 48 h post-infection (hpi), cells were fixed with formalin and immunostained using an IDV-NP polyclonal antibody (green) and 4′,6-diamidino-2-phenylindole (DAPI, blue). Representative microscopy images showed infected and control cultures in bovine (D), swine (E), canine (F), and human (H and G) primary respiratory epithelial cells. The scale bar, 400 μm. Viral NP expression was quantified in infected cells by calculating the percentages of influenza NP-positive cells relative to DAPI-positive cells. Results are expressed as mean ± SD from three randomly selected fields (I).

To determine the infectivity of IDV, we inoculated the isolated D/bovine/Jilin/HY11/2023 strain (abbreviated as D/HY11) into well-differentiated primary AEC cultures from multiple species. Viral replication was monitored by collecting supernatants at 24-h intervals for 96 h. To ensure precise characterization of the replication kinetics, we included the 12-h timepoint. We confirmed that D/HY11 can replicate in primary AEC cultures derived from humans, bovine, swine, and canine, with viral titres peaking at approximately 10^6^ TCID_50_/mL (Figure 1A-C). While the time to peak titre varies across cell cultures, the virus sustains high titres for up to 96 hpi in most cases. Notably, replication efficiency in canine AEC cultures was lower than in other species, a difference potentially attributable to variations in host cell receptor distribution [35].

For viral detection, we performed immunostaining using a custom-generated polyclonal antibody targeting the NP of the prototypic D/bovine/Oklahoma/660/2013 strain, as commercial IDV-specific antibodies are currently unavailable. This antibody has previously been validated for immunofluorescence assays [26]. The microscopic analysis of IDV-infected cultures revealed distinct clusters of NP-positive cells, while control cells showed no fluorescence signal (Figure 1D-H). Infected cultures were examined by high-content imaging to determine the percentage of viral NP-positive cells (Figure 1I). Collectively, these findings demonstrated that the novel IDV isolate possessed rapid replication capacity in multiple primary AEC types.

### Bovine IDV exhibits respiratory tract tropism in mammals

To assess the pathogenicity of IDV in mammalian hosts following intranasal inoculation, we examined its replicative capacity and tissue tropism. Three mammalian models, including mice, ferrets, and dogs, were challenged with the D/HY11strain. No clinical signs, body weight loss, or mortality were observed in any infected animals (Supplementary Figures 1C, 2B, 3F, and 3G). However, seroconversion occurred in all animals by 14 dpi (Supplementary Figure 1B, 2C, and 3E). Consistent with prior studies, no antigenic cross-reactivity was detected between IDV and circulating influenza A, B, or C viruses [1,3,10].

**Figure 2.**
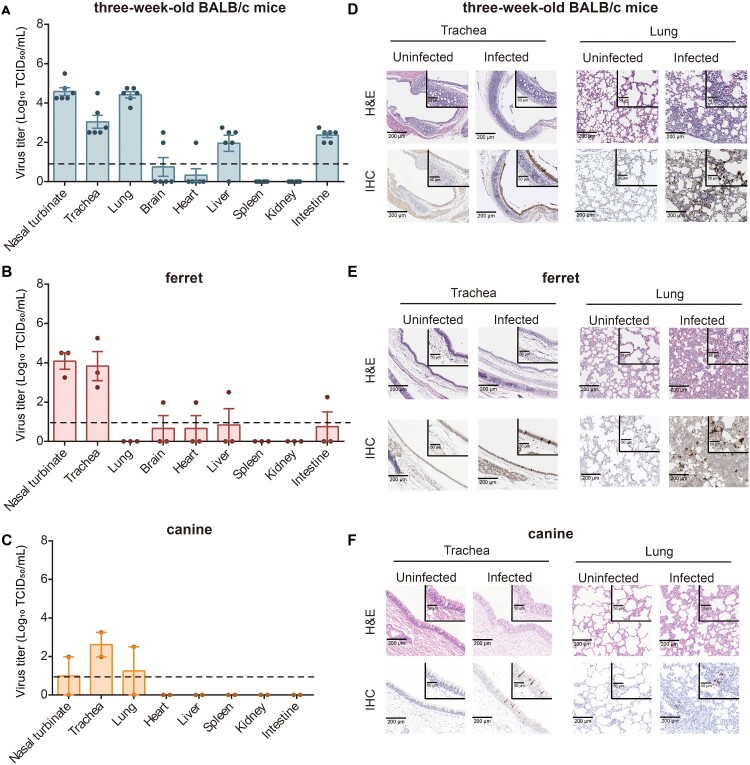
Infectivity and pathogenicity of IDV in mice, ferrets, and canines. (A) Three-week-old mice (n = 6) were intranasally inoculated with 10^6^ TCID_50_ of D/HY11. At 4 days post-infection (dpi), mice were euthanized, and viral titres in nasal turbinate, trachea, lung, brain, heart, liver, spleen, kidney, and intestine were quantified by TCID_50_ assay. (B) Ferrets (n = 3) were euthanized at 3 dpi, and viral titres in the indicated tissues (nasal turbinate, trachea, lung, brain, heart, liver, spleen, kidney, and intestine) were measured by TCID_50_ assay. (C) Dogs (n = 2) were euthanized at 3 dpi, and viral loads in nasal turbinate, trachea, lung, heart, liver, spleen, kidney, and intestine were assessed by TCID_50_ assay. Each dot represents an individual animal and data are presented as mean log_10_TCID_50_/mL ± SD. Dashed lines indicate the lower limit of virus detection. (D-F) Representative histopathology (H&E, upper panels) and immunohistochemical staining (IHC, lower panels) of trachea and lungs from mice (D), ferrets (E), and canines (F) at the specified timepoints. IDV-infected lungs displayed alveolar septal thickening and mild inflammation. Viral NP antigen was prominently localized in tracheal epithelial cells and alveoli. The scale bars represent 200 and 50 μm.

In six-week-old mice, no infectious virus was recovered from respiratory tissues, though viral RNA was detectable via qPCR (Supplementary Figure 1A). In contrast, three-week-old mice exhibited systemic viral replication, with peak titres in respiratory tracts at 4 dpi (Figure 2A, Supplementary Figure 2A). Histopathological examination revealed thickened alveolar septa with inflammatory infiltration and vascular congestion in the lung tissues of all infected animals at 4 dpi, accompanied by inflammatory cell accumulation adjacent to the tracheal epithelium. IHC analysis confirmed viral presence in both trachea and pulmonary tissues of these younger mice (Figure 2D).

In ferrets, high viral titres were observed in the upper respiratory tract (nasal turbinate and trachea), with sporadic detection in non-respiratory organs but no lung involvement (Figure 2B). IHC identified viral antigen in the trachea of all three ferrets and in the lungs of one animal (Figure 2E), suggesting limited pulmonary spread. These findings contrast with earlier reports of IDV infection restricted to nasal turbinate in ferrets [1].

In canine subjects, viral shedding and seroconversion were observed following inoculation. IDV RNA was detected in nasal, throat, and rectal swabs from 1 to 8 dpi (Supplementary Figure 3A–3C). qPCR analysis confirmed viral RNA presence in multiple tissues at 3 dpi, although measurable viral titres were restricted to respiratory tissues (Figure 2C; Supplementary Figure 3D). Histopathology examination revealed no significant tissue damage, with only sporadic IHC-positive cells detected in tracheal and pulmonary tissues (Figure 2F).

Our findings revealed notable species-specific differences in IDV infection dynamics. Despite similar viral loads, completely different pathological outcomes may occur, suggesting that host-specific factors, such as receptor distribution or innate immune responses, likely modulate IDV pathogenicity.

### IDV transmits efficiently between ferrets through the air

**Figure 3.**
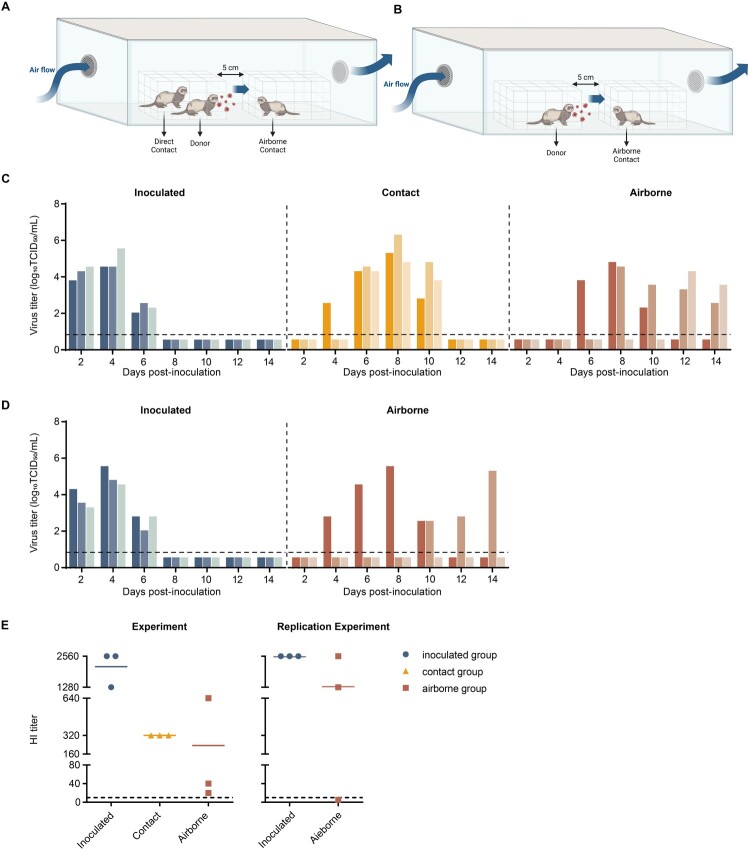
Direct contact and airborne transmission of IDV in ferrets. (A) Ferrets (n = 3) were intranasally (i.n.) inoculated with 10^6^ TCID_50_ of the D/HY11 strain. One day later, each infected ferret was co-housed with a direct-contact ferret in the same cage, and an airborne-contact ferret in an adjacent cage separated by a wire mesh partition to prevent physical contact while allowing airflow. (B) In the replication experiment, each inoculated ferret was paired with a naive ferret placed in an adjacent cage at 24 hpi to assess airborne transmission. (C and D) Nasal washes were collected every other day beginning at 2 dpi and viral titres were determined by TCID_50_ assay in MDCK cells. Coloured bars represent viral titres from individual animals (n = 3 per group). The dashed line indicates the lower limit of detection. (E) Hemagglutination inhibition (HI) antibody titres against D/HY11 were measured in serum samples from convalescent ferrets. The error bar represents mean HI titres for each group. Non-seroconverting animals are represented by values below the detection threshold.

Horizontal transmission of IDV was assessed in ferrets (Figure 3A). Ferrets infected with D/HY11 exhibited high viral titres in nasal washes over several days, peaking at 4 dpi. Notably, D/HY11 was detected in nasal washes from all contact ferrets exposed via both direct-contact and airborne-contact routes (Figure 3C). Virus was detected in nasal washes by 4 dpi (Figure 3C), and seroconversion was confirmed at 14 dpi (Figure 3E). Similarly, virus was detected in all airborne-contact ferrets from 6 to 14 dpi, with one animal remaining positive until 12 dpi (Figure 3C). Seroconversion further confirmed successful transmission (Figure 3E). Two airborne-contact ferrets showed low HI antibody titres of IDV, possibly due to delayed transmission (6 to 12 days post-exposure).

To validate airborne transmission, we repeated the experiment with separate infected and airborne-contact groups (Figure 3B). Serum was collected at 14 dpi (infected group) and 21 dpi (airborne-contact group) to account for transmission delay. Virus was detected in nasal washes from all directly infected ferrets from 2 to 6 dpi. Two of three airborne-contact ferrets showed detectable viral shedding (Figure 3D). All animals seroconverted by 14 or 21 dpi, except one airborne-contact ferret with no viral titre (Figure 3E). No significant clinical signs, weight loss, or temperature changes were observed in either group (Supplementary Figure 4).

Bovine-derived D/HY11 strain was transmitted horizontally between ferrets via both direct-contact and airborne-contact routes, contrasting with prior reports that IDV spreads only through direct contact in ferrets and guinea pigs [1,4]. Airborne transmission efficiency was 3/3 (first experiment) and 2/3 (second experiment), giving an overall transmission rate of 5/6. The incubation period ranged from 4 to 10 days. Prolonged shedding (until 12 dpi) in one ferret suggested potential transmission from a direct-contact animal rather than the initially infected group. However, we did observe that the aerosol transmission efficiency was higher in the first experiment (3/3 in both infected and direct contact groups) compared to the repeat experiment (2/3 in infected group only). This may suggest that while increased viral load in aerosols may potentially facilitate exposure, other factors such as duration of exposure or environmental conditions might also play important roles in transmission dynamics.

To determine whether ferret-adapted mutations occur in D/HY11 virus during transmission study, sequencing was performed on nasal wash samples collected from inoculated, contact and airborne ferrets. Double substitutions (amino acid A265 T and S233N) were observed on the HEF gene, while no mutations were observed in the PB1, PB2, P3, NS and P42 genes. (Figure S5)

### Serologic evidence of IDV exposure in humans

**Figure 4.**
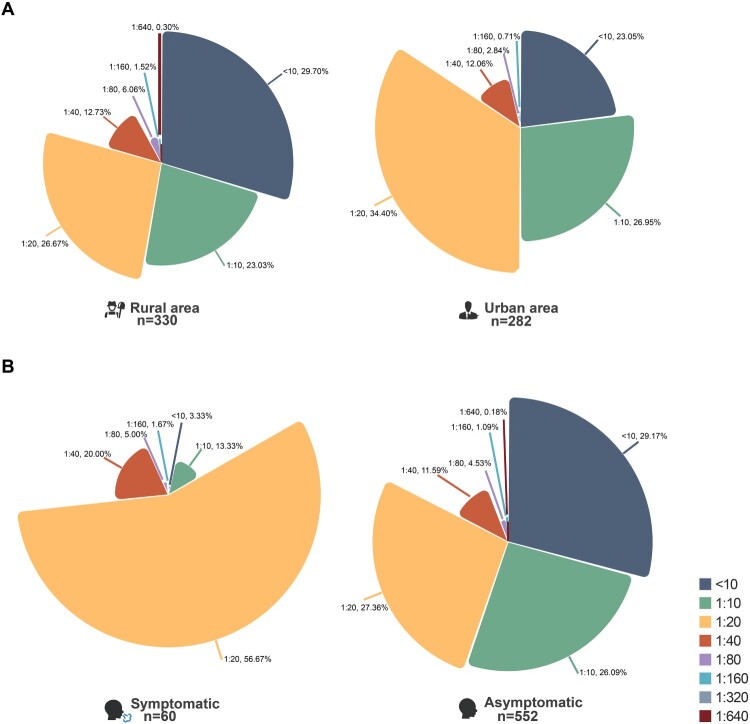
Serosurveillance results for IDV in humans in Northeast China. Proportion of HI titres by region (A) and respiratory symptoms (B).

Sera samples from 612 volunteers in Northeast China were tested for specific antibodies against D/HY11 virus using HI assays. Samples with titres equal to or higher than 10 were considered positive, with antibody titres ranging from 0 to 1:640 (Figure 4). Participants were recruited from both urban and rural areas, with a minority reporting respiratory symptoms. Serum samples collected between 2020 and 2024 were retrospectively analyzed. Demographic characteristics are detailed in Table 1. The overall seropositive rate of IDV antibodies was 73.37% (n = 612), with 18.3% showing HI titres of 1:40 or higher. No significant difference was observed between urban and rural populations. Notably, seroprevalence was significantly higher among respiratory outpatient attendees (96.67%), compared to the general healthy population (70.83%) (Figure 4B). Of 84 samples selected for MN assays (representing diverse groups and titres), HI and MN titres showed strong correlation (Figure S6). One sample with an exceptionally high HI titre (1:640) was confirmed by the MN assay. Generally, no antigenic cross-reactivity was detected between bovine IDV and human IAV/ICV [10,14].

**Table 1.** Serum testing for IDV^a^ results showing HI^b^ titres of human samples in Northeast China.

Characteristics	Seropositivity			Seronegativity
	HI titre≥10 n (%)	HI titre≥40 n (%)	HI titre≥80 n (%)	HI titre<10 n (%)
Region				
City	217 (76.95%)	44 (15.60%)	10 (3.55%)	65 (23.05%)
Countryside	232 (70.30%)	68 (20.61%)	26 (7.88%)	98 (29.70%)
Symptomatic				
Yes	58 (96.67%)	16 (26.67%)	4 (6.67%)	2 (3.33%)
No	391 (70.83%)	96 (17.39%)	32 (5.80%)	161 (29.17%)
Collection time (Year)				
2020	35 (100.00%)	11 (31.43%)	4 (11.43%)	0 (0.00%)
2021	100 (70.92%)	23 (16.31%)	3 (2.13%)	41 (29.08%)
2022	59 (72.84%)	5 (6.17%)	3 (3.70%)	22 (27.16%)
2023	23 (92.00%)	5 (20.00%)	0 (0.00%)	2 (8.00%)
2024	232 (70.30%)	68 (20.61%)	26 (7.88%)	98 (29.70%)
Total	449 (73.37%)	112 (18.30%)	36 (5.88%)	163 (26.63%)

^a^ IDV, influenza D virus.

^b^ HI, hemagglutination inhibition assay.

Samples were tested against the D/HY11 strain. For calculation purposes, titres below the detectable threshold of 10 are expressed as < 10.

### RNA polymerase inhibitors efficiently suppress IDV replication in vitro

**Figure 5.**
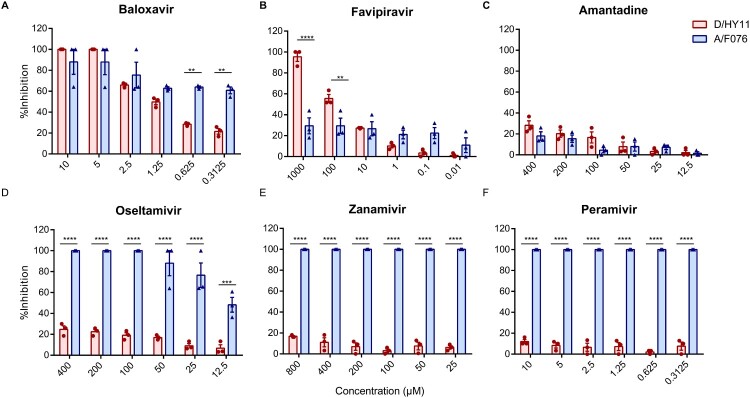
Susceptibility of influenza D and A viruses to anti-influenza drugs. (A-F) Drug susceptibility profiles of baloxavir (A), favipiravir (B), amantadine (C), oseltamivir (D), zanamivir (E), and peramivir (F) were assessed using a viral yield reduction assay in MDCK cells. Cells were infected with IDV and IAV (MOI = 0.1), followed by removal of the virus inoculum and treatment with serially diluted drugs. Supernatants were collected at designated timepoints post-infection for TCID_50_ titration. Antiviral activity (%) was calculated relative to virus-free and drug-free controls. Data represent the mean ± SD of three independent experiments. Statistical differences between D/HY11 (IDV) and A/F076 (IAV) were determined by two-way ANOVA with Bonferroni’s multiple comparisons tests (*p < 0.05, **p < 0.01, ***p < 0.001, and ****p < 0.0001).

To evaluate the efficacy of common anti-influenza drugs against IDV, we tested six compounds in a virus yield reduction assay. We used baloxavir acid (the active form of baloxavir), favipiravir, amantadine, oseltamivir acid (the active form of oseltamivir), zanamivir, and peramivir in the experiments. A human seasonal influenza A(H1N1) virus (A/Hebei/F076/2018) served as a control. D/HY11 exhibited high susceptibility to RNA polymerase inhibitors (baloxavir and favipiravir), but showed no significant sensitivity to neuraminidase inhibitors (oseltamivir, zanamivir, and peramivir) or amantadine (Figure 5 A-F). Both baloxavir and favipiravir inhibited D/HY11 replication in a dose-dependent manner, with baloxavir completely suppressing viral replication at concentrations of 5 and 10 µM. These findings suggest that RNA polymerase inhibitors may be a viable therapeutic option for IDV infection. However, further studies using a broader panel of IDV strains are needed to fullly assess drug susceptibility.

### Single gene substitutions in P3 enhance viral polymerase activity

Mutations that alter ribonucleoprotein (RNP) polymerase activity represent a well-documented adaptive strategy for influenza virus transmission in humans and mammals [36]. To determine the relative contributions of PB1, PB2, and P3 to RNP polymerase activity, mini-genome replication assays were performed in HEK-293 T cells. Notably, P3 of D/HY11 exhibited the highest polymerase activity among the tested viral polymerase complexes. Introducing single amino acid substitutions in P3 increased polymerase activity by 1.5-fold compared to the viral polymerase complexes from D/swine/Oklahoma/1334/2011, a strain that appears to be not efficient in airborne transmission in ferrets (Figure S7). The influenza virus polymerase is a key determinant of host adaptation, increased polymerase activity may promote viral pathogenicity and airborne transmission in mammals [36–38]. Our findings, while helping explain the observed phenotypic differences between strains, represent just one of several contributing factors rather than a definitive explanation for the observed tropism.

## Discussion

The recently identified IDV strain, D/HY11, replicates efficiently in human primary respiratory epithelial cells, infects mammalian hosts, transmits through the air among ferrets, and exhibits high seropositivity in humans in Northeast China. These findings suggest that D/HY11 may pose a zoonotic risk to humans and have epidemic potential. In this study, we assessed the host range of D/HY11 by examining viral replication in multiple cell types and animal species.

The limited literature available on IDV pathogenesis in vivo has suggested respiratory tropism as the primary infection pattern [1,4,39]. Our findings substantiate this respiratory tropism of D/HY11 (D/Yama2019- like clade) in mammalian hosts. Specifically in BALB/c mice, D/HY11 demonstrated predominant replication in the respiratory tract, peaking at 4 dpi. We also observed replication to a lower extent in extra-respiratory organs (liver and intestinal tissues), a phenomenon previously reported in DBA/2 mice that demonstrate enhanced permissiveness to non-adapted strains [40]. Contrary to this model, D/HY11 infected BALB/c mice without requiring prior adaptation while exhibiting greater virulence, as evidenced by both higher viral titres and prolonged shedding duration. These observations may suggest evolutionary acquisitions favouring cross-species adaptability. Although viral titres were detected in brain tissue, no pathological evidence of neurological damage was observed, leaving it unclear whether IDV could induce neurological symptoms like other influenza viruses.

We detected viral titres of IDV in the trachea of ferrets for the first time. Moreover, IHC staining revealed localized viral-positive cells in one ferret, suggesting that the infection of at least this ferret had penetrated into the lungs (Figure 2E). This contrasts with previous reports of experimental IDV infections in ferrets, where replication was limited to the upper respiratory tract and did not extend to the lungs [1].

In a recent study, IDV antibodies were detected in canines [15]. Here, we confirmed IDV infection in these companion animals and demonstrate its respiratory tropism. Furthermore, IDV was identified in rectal swabs from goats in the earlier research, indicating possible enteric tropism or oral transmitted in other susceptible species [41]. Our study also detected viral RNA in the feces of experimentally infected canines, suggesting multiple potential routes of viral shedding. Unfortunately, since no rectal swabs from cattle or swine have been tested in the field to date, it remains unclear whether IDV exhibits enteric tropism in these animals.

In the present study, experimentally infected mammals did not exhibit overt clinical signs, yet histological examination confirmed viral infection in respiratory tissues. This discrepancy may be attributed to the subclinical nature of IDV infection. In mice, prominent pulmonary lesions, including inflammatory infiltration and alveolar septal thickening, were observed, suggesting robust tissue-level responses to IDV infection despite the absence of systemic illness. Ferrets displayed localized pulmonary pathology accompanied by detectable IHC-positive signals, indicating focal viral replication and associated tissue damage. In contrast, dogs showed minimal histopathological alterations and only sporadic IHC positivity, implying lower susceptibility or more effective control of IDV replication in this species. The gradation in pathological severity – from marked changes in mice to mild or negligible lesions in dogs – may reflect intrinsic differences in host susceptibility, immune responses, or cellular tropism of IDV. The absence of clinical manifestations, despite detectable viral replication, raises concerns about the potential for asymptomatic carriers to silently transmit IDV within and between species. Further research is needed to elucidate the mechanisms driving these interspecies variations and their implications for zoonotic potential.

In particular, our study provides the first evidence of efficient airborne transmission of non-adapted bovine IDV among ferrets. While early IDV strains have been reported as non-transmissible through the air [1], our independent replicate experiments confirmed that D/HY11 has acquired airborne transmissibility. Although the virus did not cause severe clinical symptoms in any of the tested mammals and infections resolved within days, our findings underscore the potential zoonotic risk of IDV and highlight the need for increased attention of this virus. If IDV evolves to become more pathogenic, our preliminary data suggest it would likely remain susceptible to polymerase inhibitors -consitent with previous reports [32,42] while exhibiting resistance to both amantadine and neuraminidase inhibitors. A comprehensive conservation analysis of drug-binding sites across representative IDV strains from all known lineages would be crucial for elucidating the molecular basis of drug efficacy and guiding the development of broad-spectrum anti-IDV therapeutics.

Subsequent analyses revealed that IDVs exhibit higher evolutionary rate and widespread circulation [43]. Over the past decade, IDV strains have diversified from the original D/OK and D/660 lineages to include D/Yama2016, CA2019 [7,44], and, most recently, the dominant D/Yama2019 lineage, which has become prevalent in Asia [9,10]. The wider geographic distribution and expanding host range of recent IDVs, coupled with their capacity of airborne transmission, may have facilitated their ability to spill over into humans. Apart from a 2016 study of Florida occupational livestock contact workers (94%) [3], no other occupational or general population studies have reported similarly high IDV seroprevalence. Unlike previous serosurveys focused on cattle-exposed groups, our study was not limited to cattle farmers, but included individuals from both urban and rural areas of northeastern China, most of whom were not engaged in cattle farming, though their exposure history is unclear. Even though the zoonotic potential of IDV remains uncertain, the high seropositivity (73.37%) and elevated antibody titres (maximum titre 1:640) against D/HY11 in northeastern China are striking, far exceeding the previously reported IDV seropositivity was only 1.3% in the US (until 2014) [1] and 23.8% in Italy (2005–2017) [31]. Notably, seropositivity reached 96.67% among individuals with respiratory symptoms. While previous research found no statistical difference in symptom prevalence between seropositive and negative groups [45], our data suggest a potential association between IDV exposure and respiratory symptoms under natural conditions. This raises the possibility of cryptic transmission in humans with mild or asymptomatic infections via the emerging D/HY11-like viruses. Our retrospective serum analyses (2020–2024) indicate IDV may have been circulating in northeastern China since at least 2020, highlighting transmission risks during respiratory illness seasons. Currently, no routine IDV testing is carried out anywhere in the world, facilitating concerns about the silent spread of this panzootic virus and the potential emergence of new varieties.

In summary, it is likely that IDV outbreak has metastasized into an ongoing problem for cattle and humans. Unobserved subclinical infections could be important in transmission, silently sustaining epidemics at the population level. The possibility of unseen chains of transmission may silently spread through cattle, other farm animals, and humans. Close biosecurity and active surveillance in affected areas are critical to monitor any changes in outbreak dynamics.

## Limitations of the study

In our study, the unavailability of historical IDV strains and lack of reverse genetic techniques prevented us from identifying the specific adaptive mutations that facilitate sustained mammal-to-mammal transmission. Further exploration is clearly warranted. Nevertheless, our results clearly demonstrate that such transmission has been occurring silently and could be overlooked easily. Unfortunately, we were unable to find evidence of human infection with IDV at the viral level or to isolate the virus. This could be attributed to IDV’s lack of severe clinical symptoms, short detoxification period, and transient nature (as confirmed in animal infection experiments), making monitoring and isolation challenging. Consequently, despite a significant presence of positive antibodies in human serum, isolating the virus remains difficult, as our earlier research has shown [23]. This challenge may also stem from insufficient quantities of samples; hence, additional monitoring and observation are necessary.

## Ethics statement

All virus-related experiments were approved by the Institutional Biosecurity Committee and conducted under biosafety level 2 conditions. Animal studies complied with the guidelines of the Animal Welfare and Ethics Committee of the Changchun Veterinary Research Institute, Chinese Academy of Agricultural Sciences (IACUC of AMMS-11-2025-007). Housing facilities satisfied National Standards of Laboratory Animal Require­ments (GB 14925-2010, China). This study was approval by the Ethics Committee of the the Second Hospital of Jilin University (Approval No. 2024-533). Informed consent was waived by our Institutional Review Board because of the retrospective nature of our study.

## Supplementary Material

Revised Supplementary Appendix.docx
