# Supplementary material for: Efficient airborne transmission of influenza D virus in ferret models and serological evidence of human exposure in Northeast China
Source: Emerg Microbes Infect. 2025 Oct 10;14(1):2564308. doi: 10.1080/22221751.2025.2564308 (PMC12517421; doi:10.1080/22221751.2025.2564308)
Supplement: Revised Supplementary Appendix.docx [file TEMI_A_2564308_SM3200.docx]

**Supplementary Table 1. Details on primary AEC^a^ cultures.**

| Primary AEC cultures | Abbreviation | Source | Catalogue Numbers |
| --- | --- | --- | --- |
| Human nasal epithelial cells | HNECs | Cellverse Bioscience Technology Co., Ltd. | HUM-iCell-m018 |
| Human tracheal epithelial cells | HTEpic | Cellverse Bioscience Technology Co., Ltd. | HUM-iCell-a005 |
| Human alveolar epithelial cells | HAECs | Cellverse Bioscience Technology Co., Ltd. | HUM-iCell-a002 |
| Bovine nasal epithelial cells | BNECs | Cellverse Bioscience Technology Co., Ltd. | COW-iCell-m024 |
| Bovine tracheal epithelial cells | BTEpics | Cellverse Bioscience Technology Co., Ltd. | COW-iCell-a008 |
| Swine nasal epithelial cells | SNECs | Cellverse Bioscience Technology Co., Ltd. | PIG-iCell-m024 |
| Swine tracheal epithelial cells | STEpics | Cellverse Bioscience Technology Co., Ltd. | PIG-iCell-a008 |
| Canine nasal epithelial cells | CNECs | Cellverse Bioscience Technology Co., Ltd. | DOG-iCell-m024 |
| Canine tracheal epithelial cells | CTEpics | Cellverse Bioscience Technology Co., Ltd. | DOG-iCell-a008 |

^a^AEC, airway epithelial cell.





**Supplementary Figure 1. Infectivity of IDV in six-week-old mice.** At 3, 5, and 7 dpi, three mice per day were euthanized, and nasal turbinate and lung tissues were collected. The IDV RNA load in the tissues was quantified by using qPCR. Each dot represents one mouse (A). Seroconversion in infected mice at 3,5,7, and 14 days post-infection with viral inoculum (B). Body weight changes in six-week-old mice after inoculation with IDV (C).

**
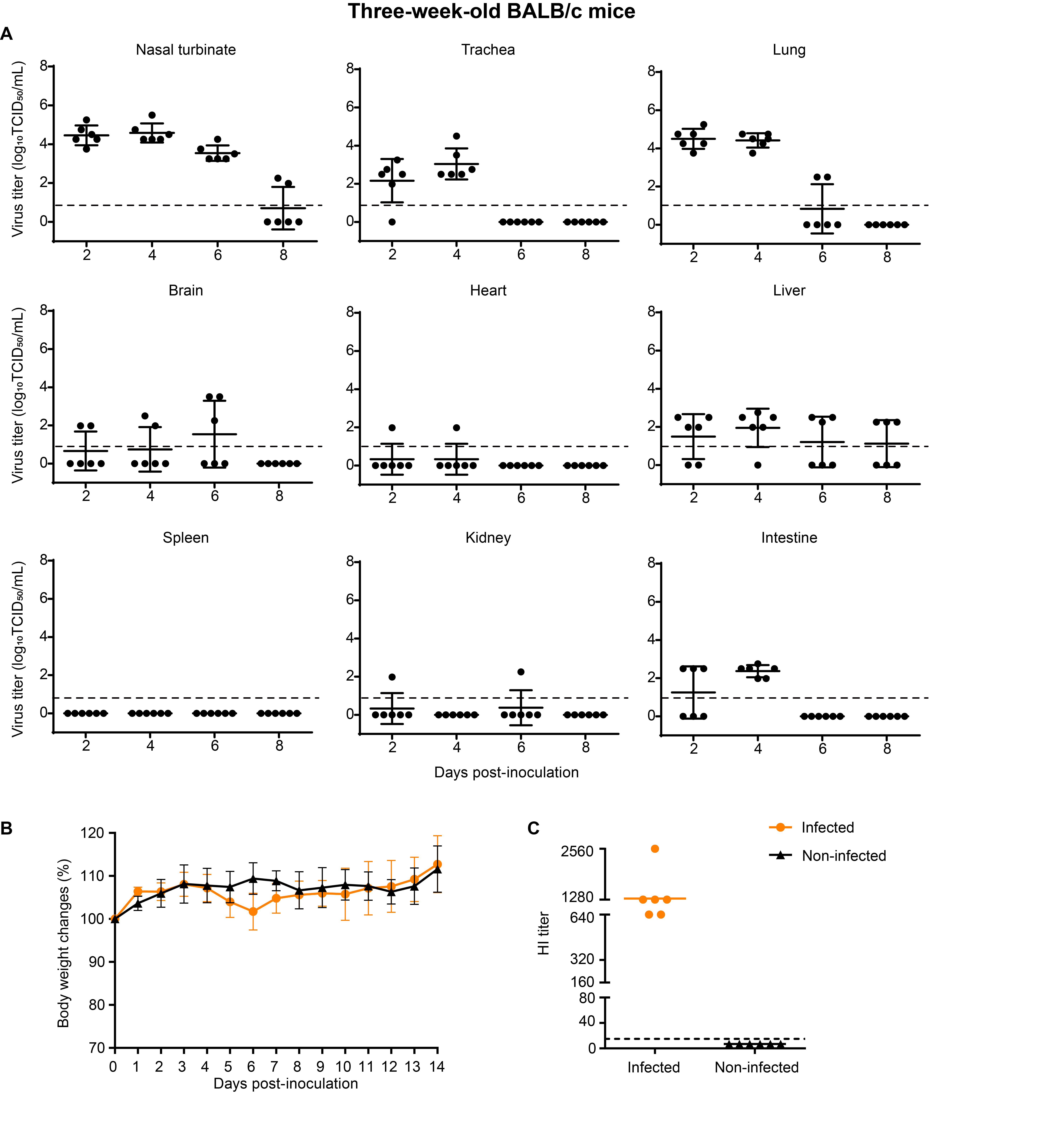
**

**Supplementary Figure 2. Systemic tropism of IDV in three-week-old mice.** Mice were infected with 10^6^ TCID_50_ of D/HY11. At 2, 4, 6, and 8 dpi, six mice per day were euthanized, and nasal turbinate, trachea, lung, brain, heart, liver, spleen, kidney, and intestine samples were collected. Virus titers were determined using the TCID_50_ method. Dashed lines indicate the lower limit of virus detection. Each dot represents one mouse (A, related to Figure 2A**)**. Body weight changes in mice after inoculation with IDV. Body weight was recorded every day for 14 days. There was no significant weight variation between non-infected and infected mice (*P* > 0.05). Weight changes are expressed as the mean percentage of the initial body weight ± SD (B). Sera collected from infected and non-infected mice at 14 dpi were subjected to HI assays with D/HY11, and HI titers are shown. The floating bars represent the mean HI titer for each group. Mice showing no seroconversion were assigned arbitrary values below the limit of detection so they could be represented on the graph (C).


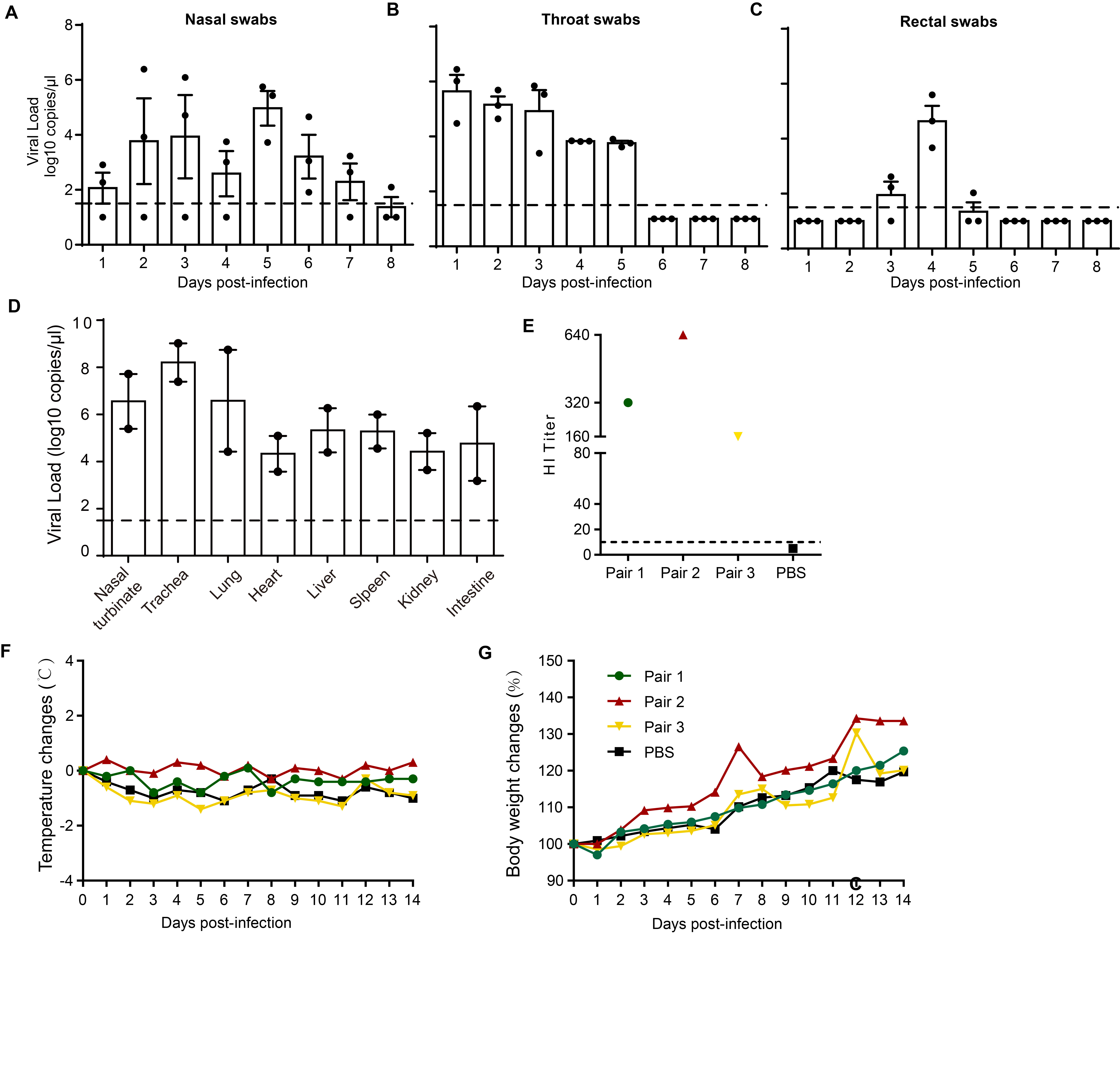


**Supplementary Figure 3. IDV infectivity in canines.** IDV RNA load in nasal (A), throat (B), and rectal (C) swab samples quantified using qPCR at 1 to 8 dpi; viral RNA was not detected in any of the control animals (data not shown). At 3 dpi, other dogs (n = 2) were euthanized, and nasal turbinate, trachea, lung, heart, liver, spleen, kidney, and intestine tissues were collected. Virus RNA load was quantified using qPCR. Each dot represents one dog (D, related to figure 2C). Seroconversion in infected canines at 14 dpi with viral inoculum (D/HY11); none of the non-infected canines seroconverted (E). Body weight (F) and temperature (G) changes in dogs. Dashed lines indicate the lower limit of virus detection.

**
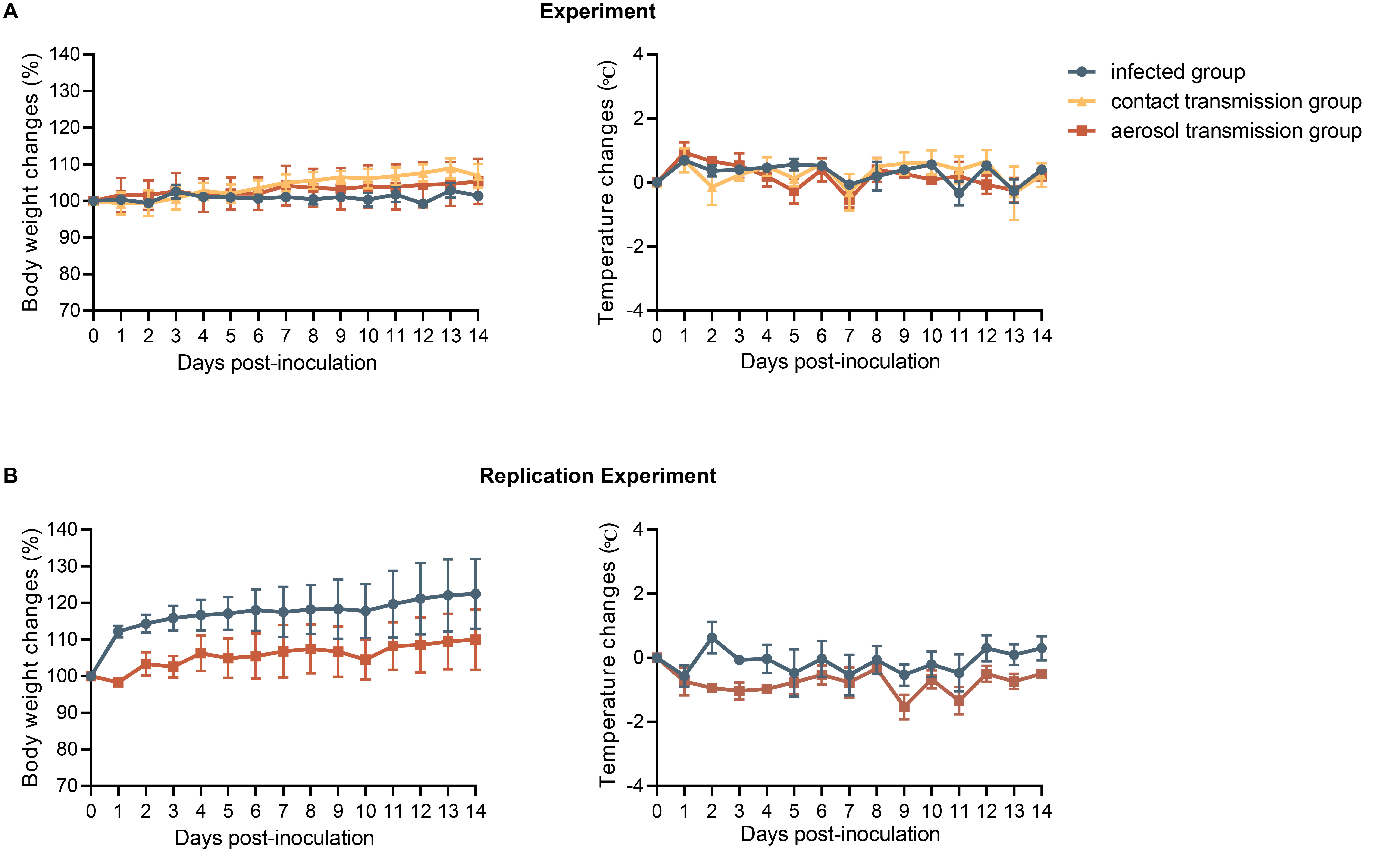
**

**Supplementary Figure 4. Clinical data associated with ferrets used to assess airborne transmission.** Body weight and temperature changes in ferrets used in the transmission experiment (A) and the independent replication experiment (B).

**
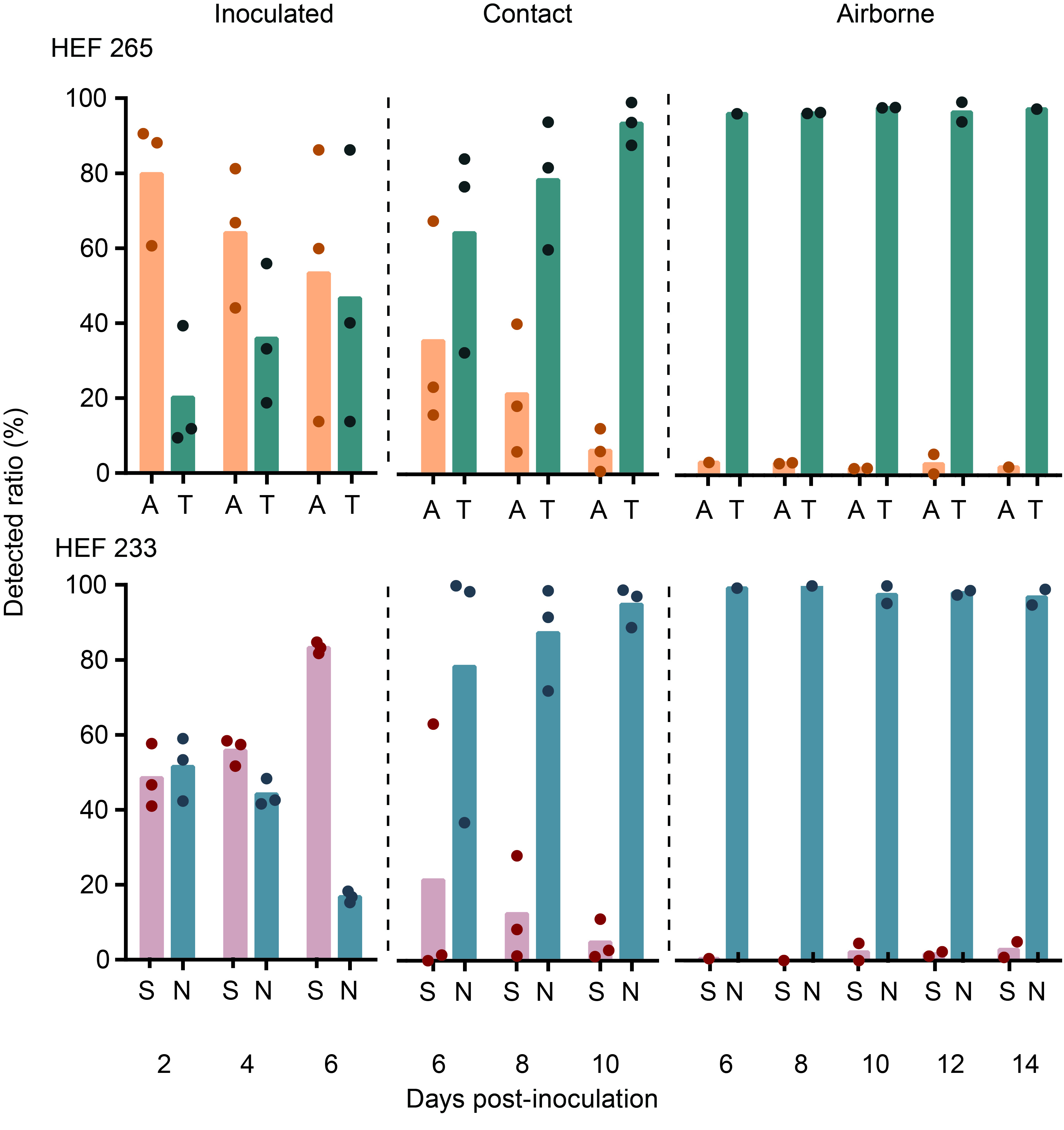
**

**Supplementary Figure 5 Progressive sequence changes of virus in ferrets in transmission study.**

Dynamic mutation frequencies of different amino acids at HEF-265 and 233 during replication and transmission of D/HY11 virus in ferrets. Each circle represents the detected ratio from an individual animal. Columns are mean frequencies.


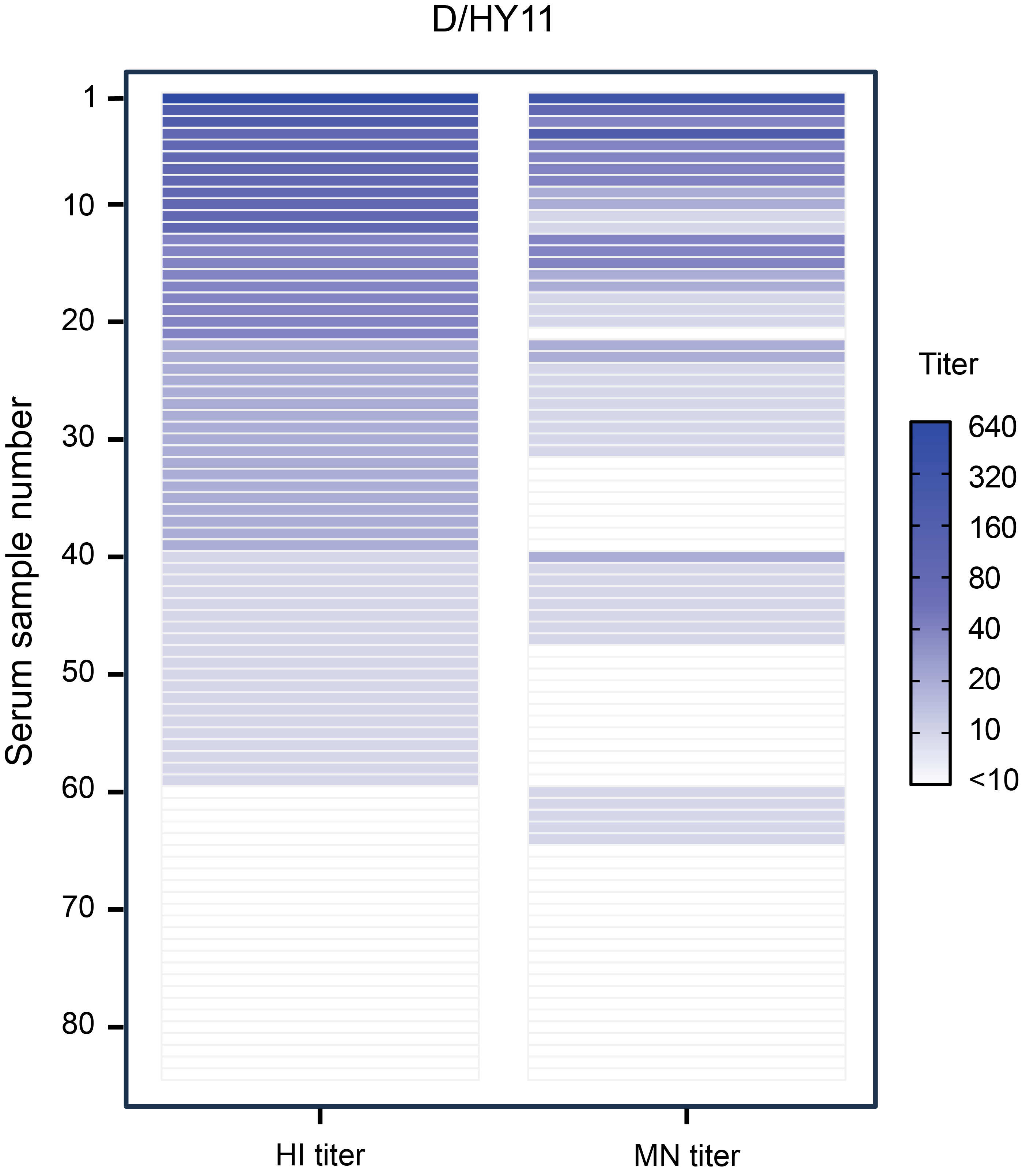


## Supplementary Figure 6. HI and MN antibodies against IDV in 84 serum samples obtained from humans.

HI (left) and MN (right) antibodies against D/HY11 in 84 of 612 serum samples obtained from humans. Rectangular boxes filled with blue colors indicate HI or MN antibody titers. Titers below the detectable threshold of 10 are expressed as <10. The blank rectangular boxes represent serum samples with no detectable HI or MN antibody titers.


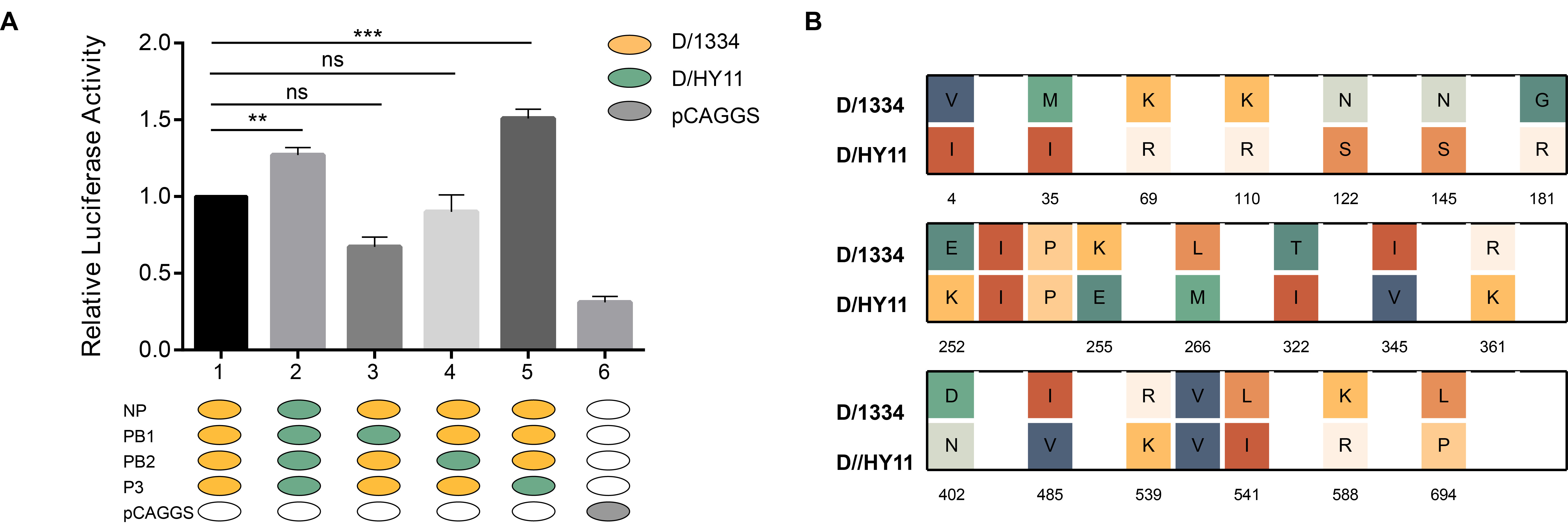


**Supplementary Figure 7. Effect of single gene substitutions in PB1, PB2, and P3 on RNA polymerase complex activity.**

(A) Relative polymerase activity of single-gene reassorted RNPs compared to D/1334. Plasmids encoding the PB2, PB1, P3, and NP proteins from D/1334 and D/HY11 with different combinations were transfected into HEK293T cells along with the firefly luciferase reporter plasmid (p-NP-Luci) and an internal control plasmid (pRL-TK). The negative control consisted of p-NP-Luci, pRL-TK, and the empty vector plasmid (pCAGGS) without influenza virus genes. Luciferase activity was measured 32 hours post-transfection. Data are presented as mean±*SD*. RNA polymerase activity was analyzed using one-way ANOVA; asterisks indicate statistically significant differences between groups (**p* < 0.05, ***p*<0.01, and ****p*<0.001). "ns" denotes no significant difference. (B) Sequence alignment of key residues in the P3 protein from representative IDV strains. D/Jilin/HY11/2023 abbreviated as D/HY11, and D/swine/Oklahoma/1334/2011 abbreviated as D/1334.
